# 2,2′-[1-(2,4,6-Trichlorophenyl)-1*H*-1,2,4-triazole-3,5-diyl]diphenol

**DOI:** 10.1107/S1600536808001505

**Published:** 2008-01-23

**Authors:** Zhong-Shu Li, Xiu-Bing Li, Bai-Wang Sun

**Affiliations:** a Ordered Matter Science Research Center, College of Chemistry and Chemical Engineering, Southeast University, Nanjing 210096, People’s Republic of China; b Department of Chemistry, Key Laboratory of Medicinal Chemistry for Natural Resources of the Ministry of Education, Yunnan University, Kunming 650091, People’s Republic of China

## Abstract

The title compound, C_20_H_12_Cl_3_N_3_O_2_, was synthesized by the reaction of 2-(2-hydroxy­phen­yl)benz[*e*][1,3]oxazin-4-one with 2,4,6-trichloro­phenyl­hydrazine in ethanol. The trichloro­phenyl ring is nearly perpendicular to the triazole plane [dihedral angle 80.56 (8)°], whereas the two hydroxy­phenyl rings are approximately coplanar with the triazole ring [dihedral angles of 2.79 (12) and 8.00 (14)°]. Intra­molecular O—H⋯N hydrogen bonding is observed between the hydroxy­phenyl and triazole rings.

## Related literature

For general background, see: Nisbet-Brown *et al.* (2003 ▶); Steinhauser *et al.* (2004 ▶).
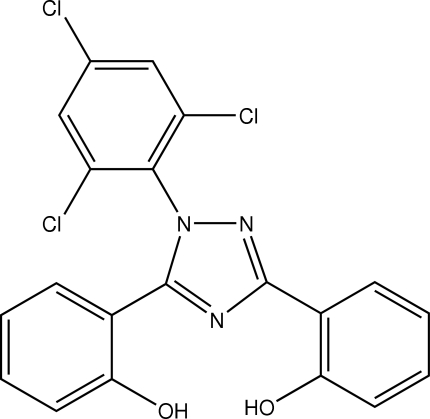


## Experimental

### 

#### Crystal data

C_20_H_12_Cl_3_N_3_O_2_
*M* *_r_* = 432.68
Monoclinic, 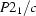

*a* = 14.328 (3) Å
*b* = 12.021 (2) Å
*c* = 12.014 (2) Å
β = 104.99 (3)°
*V* = 1998.7 (7) Å^3^
*Z* = 4
Mo *K*α radiation
μ = 0.48 mm^−1^
*T* = 293 (2) K
0.20 × 0.20 × 0.18 mm

#### Data collection

Rigaku SCXmini diffractometer
Absorption correction: multi-scan (*CrystalClear*; Rigaku, 2005 ▶) *T*_min_ = 0.907, *T*_max_ = 0.915
16254 measured reflections
3501 independent reflections
3052 reflections with *I* > 2σ(*I*)
*R*_int_ = 0.047

#### Refinement

*R*[*F* ^2^ > 2σ(*F* ^2^)] = 0.044
*wR*(*F* ^2^) = 0.104
*S* = 1.11
3501 reflections
261 parameters
H atoms treated by a mixture of independent and constrained refinement
Δρ_max_ = 0.35 e Å^−3^
Δρ_min_ = −0.35 e Å^−3^

### 

Data collection: *CrystalClear* (Rigaku, 2005 ▶); cell refinement: *CrystalClear*; data reduction: *CrystalClear*; program(s) used to solve structure: *SHELXTL/PC* (Sheldrick, 2008 ▶); program(s) used to refine structure: *SHELXTL/PC*; molecular graphics: *SHELXTL/PC*; software used to prepare material for publication: *SHELXTL/PC*.

## Supplementary Material

Crystal structure: contains datablocks I, global. DOI: 10.1107/S1600536808001505/xu2398sup1.cif

Structure factors: contains datablocks I. DOI: 10.1107/S1600536808001505/xu2398Isup2.hkl

Additional supplementary materials:  crystallographic information; 3D view; checkCIF report

## Figures and Tables

**Table 1:** Hydrogen-bond geometry (Å, °)

*D*—H⋯*A*	*D*—H	H⋯*A*	*D*⋯*A*	*D*—H⋯*A*
O1—H1*A*⋯N3	0.83 (3)	1.89 (3)	2.640 (3)	149 (3)
O2—H2*A*⋯N2	0.81 (2)	1.94 (2)	2.648 (3)	146 (3)

**Fig. 1.**
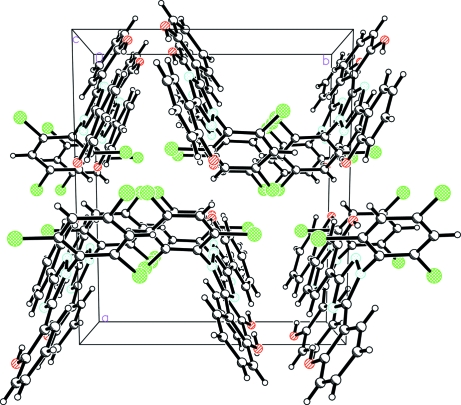
The molecular structure of the title molecule, with the atom-numbering scheme. Displacement ellipsoids are drawn at the 30% probability level.

## supplementary crystallographic information

### Comment

3,5-Bis(2-hydroxyphenyl)-1-phenyl-1,2,4-triazole core has been successfully used a motif for the development of biologically interesting molecules, including active iron chelator (Nisbet-Brown *et al.*, 2003; Steinhauser *et al.*, 2004). We report here the crystal structure of the title triazole compound.

In the title molecule (Fig. 1), 3-(2-hydroxyphenyl) is almost co-planar with 1,2,4-triazole ring, dihedral angle being 2.79 (12)°. The 5-(2-hydroxyphenyl) ring forms a dihedral angle of 9.70 (13)° with triazole plane. The trichlorophenyl is nearly perpendicular to the triazole plane with a dihedral angle of 80.56 (8)°. Intra-molecular N—H···O hydrogen bonding is observed between hydroxyphenyl and triazole rings (Table 1).

### Experimental

2-(2-Hydroxyphenyl)benz[*e*][1,3]oxazin-4-one (2.4 g) was mixed with 2,4,6-trichlorophenylhydrazine (2.2 g) in ethanol (30 ml). The mixture was refluxed for 3 h, after cooling to room temperature, the mixture was poured onto water and extracted with ethyl acetate. The combined organic phases were dried over sodium sulfate and concentrated on a rotary evaporator. The title compound was crystallized from methanol. The colourless crystals were obtained by slow evaporation of methanol.

### Refinement

H atoms bound to carbon were placed in calculated positions and refined in riding mode with C—H = 0.93 Å and *U*_iso_(H)=1.2*U*_eq_(C). Hydroxyl H atoms were located in a difference Fourier map and refined isotropically.

### Crystal data

**Table:**

C_20_H_12_Cl_3_N_3_O_2_	*F*(000) = 880
*M**_r_* = 432.68	*D*_x_ = 1.438 Mg m^−^^3^
Monoclinic, *P*2_1_/*c*	Mo *K*α radiation, λ = 0.71073 Å
Hall symbol: -P 2ybc	Cell parameters from 5847 reflections
*a* = 14.328 (3) Å	θ = 3.0–28.4°
*b* = 12.021 (2) Å	µ = 0.48 mm^−^^1^
*c* = 12.014 (2) Å	*T* = 293 K
β = 104.99 (3)°	Block, colourless
*V* = 1998.7 (7) Å^3^	0.20 × 0.20 × 0.18 mm
*Z* = 4	

### Data collection

**Table:**

Rigaku SCXmini diffractometer	3501 independent reflections
Radiation source: fine-focus sealed tube	3052 reflections with *I* > 2σ(*I*)
graphite	*R*_int_ = 0.047
Detector resolution: 8.192 pixels mm^-1^	θ_max_ = 25.0°, θ_min_ = 3.1°
ω scans	*h* = −17→17
Absorption correction: multi-scan (*CrystalClear*; Rigaku, 2005)	*k* = −14→14
*T*_min_ = 0.907, *T*_max_ = 0.915	*l* = −14→14
16254 measured reflections	

### Refinement

**Table:**

Refinement on *F*^2^	Primary atom site location: structure-invariant direct methods
Least-squares matrix: full	Secondary atom site location: difference Fourier map
*R*[*F*^2^ > 2σ(*F*^2^)] = 0.044	Hydrogen site location: inferred from neighbouring sites
*wR*(*F*^2^) = 0.104	H atoms treated by a mixture of independent and constrained refinement
*S* = 1.11	*w* = 1/[σ^2^(*F*_o_^2^) + (0.034*P*)^2^ + 0.892*P*] where *P* = (*F*_o_^2^ + 2*F*_c_^2^)/3
3501 reflections	(Δ/σ)_max_ < 0.001
261 parameters	Δρ_max_ = 0.36 e Å^−^^3^
0 restraints	Δρ_min_ = −0.35 e Å^−^^3^

### Special details

**Table:**

Geometry. All e.s.d.'s (except the e.s.d. in the dihedral angle between two l.s. planes) are estimated using the full covariance matrix. The cell e.s.d.'s are taken into account individually in the estimation of e.s.d.'s in distances, angles and torsion angles; correlations between e.s.d.'s in cell parameters are only used when they are defined by crystal symmetry. An approximate (isotropic) treatment of cell e.s.d.'s is used for estimating e.s.d.'s involving l.s. planes.
Refinement. Refinement of *F*^2^ against ALL reflections. The weighted *R*-factor *wR* and goodness of fit *S* are based on *F*^2^, conventional *R*-factors *R* are based on *F*, with *F* set to zero for negative *F*^2^. The threshold expression of *F*^2^ > σ(*F*^2^) is used only for calculating *R*-factors(gt) *etc*. and is not relevant to the choice of reflections for refinement. *R*-factors based on *F*^2^ are statistically about twice as large as those based on *F*, and *R*- factors based on ALL data will be even larger.

### Fractional atomic coordinates and isotropic or equivalent isotropic displacement parameters (Å^2^)

**Table:**

	*x*	*y*	*z*	*U*_iso_*/*U*_eq_	
Cl1	0.25005 (6)	−0.25222 (6)	0.53114 (7)	0.0819 (3)	
Cl2	0.36388 (5)	0.15110 (5)	0.40863 (6)	0.0679 (2)	
Cl3	0.49510 (6)	−0.22786 (7)	0.25494 (7)	0.0814 (2)	
N1	0.25668 (12)	−0.00229 (15)	0.52989 (14)	0.0448 (4)	
N2	0.29844 (12)	0.00432 (15)	0.64746 (14)	0.0452 (4)	
N3	0.14978 (11)	0.08336 (15)	0.60312 (14)	0.0437 (4)	
C1	0.33274 (16)	0.04839 (17)	0.89318 (18)	0.0460 (5)	
C2	0.34437 (19)	0.0687 (2)	1.0104 (2)	0.0625 (6)	
H2C	0.4018	0.0490	1.0632	0.075*	
C3	0.2707 (2)	0.1181 (2)	1.0485 (2)	0.0678 (7)	
H3B	0.2788	0.1305	1.1268	0.081*	
C4	0.1850 (2)	0.1491 (2)	0.9706 (2)	0.0638 (7)	
H4A	0.1360	0.1823	0.9967	0.077*	
C5	0.17289 (17)	0.13019 (19)	0.8534 (2)	0.0531 (6)	
H5A	0.1155	0.1513	0.8014	0.064*	
C6	0.24623 (14)	0.07958 (17)	0.81220 (17)	0.0410 (5)	
C7	0.23151 (14)	0.05618 (16)	0.68776 (17)	0.0399 (4)	
C8	0.16669 (14)	0.04599 (17)	0.50442 (18)	0.0422 (5)	
C9	0.09851 (15)	0.06060 (19)	0.38957 (18)	0.0483 (5)	
C10	0.01411 (16)	0.1254 (2)	0.3802 (2)	0.0555 (6)	
C11	−0.04752 (19)	0.1462 (2)	0.2709 (3)	0.0749 (8)	
H11A	−0.1024	0.1895	0.2645	0.090*	
C12	−0.0282 (2)	0.1036 (3)	0.1730 (3)	0.0841 (9)	
H12A	−0.0691	0.1202	0.1012	0.101*	
C13	0.0522 (2)	0.0357 (3)	0.1803 (2)	0.0850 (9)	
H13A	0.0637	0.0045	0.1142	0.102*	
C14	0.11476 (18)	0.0154 (3)	0.2880 (2)	0.0698 (7)	
H14A	0.1687	−0.0292	0.2930	0.084*	
C15	0.31228 (14)	−0.05594 (18)	0.46145 (17)	0.0430 (5)	
C16	0.36702 (15)	0.00659 (18)	0.40185 (17)	0.0454 (5)	
C17	0.42321 (16)	−0.0452 (2)	0.33763 (19)	0.0524 (6)	
H17A	0.4587	−0.0035	0.2978	0.063*	
C18	0.42469 (17)	−0.1615 (2)	0.3349 (2)	0.0550 (6)	
C19	0.37295 (18)	−0.2260 (2)	0.3944 (2)	0.0609 (6)	
H19A	0.3758	−0.3033	0.3923	0.073*	
C20	0.31687 (16)	−0.1727 (2)	0.4570 (2)	0.0520 (5)	
O1	−0.01130 (13)	0.16967 (17)	0.47345 (18)	0.0718 (5)	
H1A	0.032 (2)	0.156 (3)	0.533 (3)	0.087 (10)*	
O2	0.40802 (12)	−0.00300 (15)	0.86177 (16)	0.0593 (4)	
H2A	0.3949 (19)	−0.013 (2)	0.793 (2)	0.064 (9)*	

### Atomic displacement parameters (Å^2^)

**Table:**

	*U*^11^	*U*^22^	*U*^33^	*U*^12^	*U*^13^	*U*^23^
Cl1	0.0879 (5)	0.0692 (5)	0.1039 (6)	−0.0208 (4)	0.0528 (5)	−0.0016 (4)
Cl2	0.0863 (5)	0.0487 (3)	0.0809 (5)	0.0037 (3)	0.0437 (4)	0.0037 (3)
Cl3	0.0916 (5)	0.0814 (5)	0.0878 (5)	0.0185 (4)	0.0529 (4)	−0.0117 (4)
N1	0.0394 (9)	0.0578 (11)	0.0380 (9)	0.0044 (8)	0.0116 (7)	−0.0019 (8)
N2	0.0411 (9)	0.0563 (11)	0.0381 (9)	0.0046 (8)	0.0100 (7)	−0.0020 (8)
N3	0.0368 (9)	0.0510 (10)	0.0449 (10)	0.0025 (7)	0.0136 (8)	0.0033 (8)
C1	0.0516 (12)	0.0416 (11)	0.0455 (12)	0.0003 (9)	0.0140 (10)	0.0031 (9)
C2	0.0686 (16)	0.0713 (16)	0.0440 (13)	0.0056 (13)	0.0083 (11)	0.0029 (11)
C3	0.091 (2)	0.0722 (17)	0.0421 (14)	0.0006 (15)	0.0210 (13)	−0.0036 (12)
C4	0.0784 (17)	0.0642 (16)	0.0581 (16)	0.0057 (13)	0.0345 (14)	−0.0086 (12)
C5	0.0543 (13)	0.0559 (14)	0.0511 (13)	0.0070 (11)	0.0176 (11)	−0.0018 (10)
C6	0.0460 (11)	0.0383 (10)	0.0411 (11)	−0.0023 (9)	0.0152 (9)	0.0008 (8)
C7	0.0383 (10)	0.0419 (11)	0.0409 (11)	−0.0005 (8)	0.0130 (9)	0.0016 (8)
C8	0.0358 (10)	0.0487 (12)	0.0438 (12)	−0.0022 (9)	0.0136 (9)	0.0033 (9)
C9	0.0389 (11)	0.0603 (14)	0.0441 (12)	−0.0057 (10)	0.0079 (9)	0.0071 (10)
C10	0.0417 (12)	0.0601 (14)	0.0614 (15)	−0.0054 (10)	0.0075 (11)	0.0078 (11)
C11	0.0527 (15)	0.0833 (19)	0.075 (2)	0.0037 (14)	−0.0075 (14)	0.0187 (15)
C12	0.0689 (19)	0.114 (3)	0.0552 (17)	−0.0103 (17)	−0.0095 (14)	0.0228 (16)
C13	0.0680 (18)	0.136 (3)	0.0463 (16)	−0.0049 (18)	0.0067 (13)	−0.0005 (16)
C14	0.0504 (14)	0.110 (2)	0.0458 (14)	0.0045 (14)	0.0061 (11)	−0.0033 (14)
C15	0.0380 (10)	0.0542 (13)	0.0371 (11)	0.0028 (9)	0.0102 (9)	−0.0043 (9)
C16	0.0453 (12)	0.0498 (12)	0.0418 (11)	0.0031 (9)	0.0124 (9)	0.0005 (9)
C17	0.0521 (13)	0.0618 (15)	0.0476 (13)	0.0048 (11)	0.0210 (10)	0.0042 (10)
C18	0.0550 (13)	0.0635 (15)	0.0506 (13)	0.0096 (11)	0.0210 (11)	−0.0079 (11)
C19	0.0673 (16)	0.0496 (13)	0.0704 (17)	0.0009 (11)	0.0262 (13)	−0.0083 (11)
C20	0.0499 (12)	0.0550 (14)	0.0537 (13)	−0.0060 (10)	0.0180 (10)	−0.0029 (10)
O1	0.0472 (10)	0.0926 (14)	0.0714 (13)	0.0185 (9)	0.0078 (9)	−0.0004 (10)
O2	0.0530 (10)	0.0742 (12)	0.0491 (10)	0.0160 (8)	0.0103 (8)	0.0052 (9)

### Geometric parameters (Å, °)

**Table:**

Cl1—C20	1.751 (2)	C9—C14	1.410 (3)
Cl2—C16	1.740 (2)	C9—C10	1.419 (3)
Cl3—C18	1.754 (2)	C10—O1	1.371 (3)
N1—C8	1.374 (3)	C10—C11	1.402 (4)
N1—N2	1.386 (2)	C11—C12	1.375 (4)
N1—C15	1.437 (2)	C11—H11A	0.9300
N2—C7	1.335 (3)	C12—C13	1.395 (4)
N3—C8	1.347 (3)	C12—H12A	0.9300
N3—C7	1.377 (3)	C13—C14	1.392 (4)
C1—O2	1.378 (3)	C13—H13A	0.9300
C1—C2	1.396 (3)	C14—H14A	0.9300
C1—C6	1.414 (3)	C15—C20	1.406 (3)
C2—C3	1.388 (4)	C15—C16	1.408 (3)
C2—H2C	0.9300	C16—C17	1.397 (3)
C3—C4	1.387 (4)	C17—C18	1.399 (3)
C3—H3B	0.9300	C17—H17A	0.9300
C4—C5	1.393 (3)	C18—C19	1.391 (3)
C4—H4A	0.9300	C19—C20	1.391 (3)
C5—C6	1.410 (3)	C19—H19A	0.9300
C5—H5A	0.9300	O1—H1A	0.83 (3)
C6—C7	1.482 (3)	O2—H2A	0.80 (3)
C8—C9	1.480 (3)		
			
C8—N1—N2	109.60 (16)	C11—C10—C9	119.3 (2)
C8—N1—C15	133.55 (17)	C12—C11—C10	121.2 (3)
N2—N1—C15	116.84 (15)	C12—C11—H11A	119.4
C7—N2—N1	103.59 (15)	C10—C11—H11A	119.4
C8—N3—C7	104.99 (16)	C11—C12—C13	120.6 (3)
O2—C1—C2	117.2 (2)	C11—C12—H12A	119.7
O2—C1—C6	122.65 (19)	C13—C12—H12A	119.7
C2—C1—C6	120.2 (2)	C14—C13—C12	118.9 (3)
C3—C2—C1	120.3 (2)	C14—C13—H13A	120.5
C3—C2—H2C	119.9	C12—C13—H13A	120.5
C1—C2—H2C	119.9	C13—C14—C9	121.8 (3)
C4—C3—C2	120.6 (2)	C13—C14—H14A	119.1
C4—C3—H3B	119.7	C9—C14—H14A	119.1
C2—C3—H3B	119.7	C20—C15—C16	118.40 (19)
C3—C4—C5	119.7 (2)	C20—C15—N1	120.51 (19)
C3—C4—H4A	120.2	C16—C15—N1	121.03 (19)
C5—C4—H4A	120.2	C17—C16—C15	121.3 (2)
C4—C5—C6	121.0 (2)	C17—C16—Cl2	119.75 (17)
C4—C5—H5A	119.5	C15—C16—Cl2	118.97 (16)
C6—C5—H5A	119.5	C16—C17—C18	118.2 (2)
C5—C6—C1	118.25 (19)	C16—C17—H17A	120.9
C5—C6—C7	120.75 (19)	C18—C17—H17A	120.9
C1—C6—C7	120.97 (18)	C19—C18—C17	122.1 (2)
N2—C7—N3	113.35 (17)	C19—C18—Cl3	119.10 (19)
N2—C7—C6	121.76 (18)	C17—C18—Cl3	118.77 (18)
N3—C7—C6	124.89 (17)	C18—C19—C20	118.7 (2)
N3—C8—N1	108.46 (17)	C18—C19—H19A	120.7
N3—C8—C9	123.73 (18)	C20—C19—H19A	120.7
N1—C8—C9	127.77 (19)	C19—C20—C15	121.3 (2)
C14—C9—C10	118.1 (2)	C19—C20—Cl1	119.44 (19)
C14—C9—C8	123.0 (2)	C15—C20—Cl1	119.25 (17)
C10—C9—C8	118.8 (2)	C10—O1—H1A	109 (2)
O1—C10—C11	117.4 (2)	C1—O2—H2A	110.6 (19)
O1—C10—C9	123.3 (2)		
			
C8—N1—N2—C7	0.1 (2)	C14—C9—C10—O1	177.2 (2)
C15—N1—N2—C7	−179.52 (17)	C8—C9—C10—O1	−4.5 (3)
O2—C1—C2—C3	−178.7 (2)	C14—C9—C10—C11	−2.7 (3)
C6—C1—C2—C3	0.9 (4)	C8—C9—C10—C11	175.6 (2)
C1—C2—C3—C4	−0.8 (4)	O1—C10—C11—C12	−179.0 (3)
C2—C3—C4—C5	0.2 (4)	C9—C10—C11—C12	0.9 (4)
C3—C4—C5—C6	0.2 (4)	C10—C11—C12—C13	1.9 (5)
C4—C5—C6—C1	0.0 (3)	C11—C12—C13—C14	−2.6 (5)
C4—C5—C6—C7	177.9 (2)	C12—C13—C14—C9	0.7 (5)
O2—C1—C6—C5	179.0 (2)	C10—C9—C14—C13	2.0 (4)
C2—C1—C6—C5	−0.6 (3)	C8—C9—C14—C13	−176.3 (3)
O2—C1—C6—C7	1.1 (3)	C8—N1—C15—C20	−100.5 (3)
C2—C1—C6—C7	−178.4 (2)	N2—N1—C15—C20	79.1 (2)
N1—N2—C7—N3	−0.2 (2)	C8—N1—C15—C16	82.6 (3)
N1—N2—C7—C6	179.23 (17)	N2—N1—C15—C16	−97.8 (2)
C8—N3—C7—N2	0.2 (2)	C20—C15—C16—C17	1.3 (3)
C8—N3—C7—C6	−179.23 (18)	N1—C15—C16—C17	178.30 (19)
C5—C6—C7—N2	−177.3 (2)	C20—C15—C16—Cl2	−178.83 (16)
C1—C6—C7—N2	0.6 (3)	N1—C15—C16—Cl2	−1.8 (3)
C5—C6—C7—N3	2.1 (3)	C15—C16—C17—C18	−0.7 (3)
C1—C6—C7—N3	179.90 (19)	Cl2—C16—C17—C18	179.45 (17)
C7—N3—C8—N1	−0.1 (2)	C16—C17—C18—C19	−0.5 (4)
C7—N3—C8—C9	−178.01 (19)	C16—C17—C18—Cl3	−179.86 (17)
N2—N1—C8—N3	0.0 (2)	C17—C18—C19—C20	1.0 (4)
C15—N1—C8—N3	179.5 (2)	Cl3—C18—C19—C20	−179.63 (19)
N2—N1—C8—C9	177.80 (19)	C18—C19—C20—C15	−0.3 (4)
C15—N1—C8—C9	−2.6 (4)	C18—C19—C20—Cl1	179.26 (19)
N3—C8—C9—C14	−176.2 (2)	C16—C15—C20—C19	−0.8 (3)
N1—C8—C9—C14	6.3 (4)	N1—C15—C20—C19	−177.8 (2)
N3—C8—C9—C10	5.6 (3)	C16—C15—C20—Cl1	179.62 (16)
N1—C8—C9—C10	−172.0 (2)	N1—C15—C20—Cl1	2.6 (3)

### Hydrogen-bond geometry (Å, °)

**Table:**

*D*—H···*A*	*D*—H	H···*A*	*D*···*A*	*D*—H···*A*
O1—H1A···N3	0.83 (3)	1.89 (3)	2.640 (3)	149 (3)
O2—H2A···N2	0.81 (2)	1.94 (2)	2.648 (3)	146 (3)
